# A national study on weight classes among children in Greenland at school entry

**DOI:** 10.3402/ijch.v73.25537

**Published:** 2014-10-20

**Authors:** Karsten F. Rex, Nicolai H. Larsen, Hanne Rex, Birgit Niclasen, Michael L. Pedersen

**Affiliations:** 1 Queen Ingrid Healthcare Center, Nuuk, Greenland; 2 National Institute of Public Health, University of Southern Denmark, Odense M, Denmark; 3 Greenland Center for Health Research, Institute of Nursing and Health Science, University of Greenland, Nuuk, Greenland

**Keywords:** Inuit, Greenland, overweight, obesity, thinness, normal weight, children, cross-sectional, BMI

## Abstract

**Objectives:**

To estimate the proportion of gender-specific thinness, overweight and obesity among children born in 2005 at school entry in Greenland and to compare figures between the capital, Nuuk, with the rest of Greenland.

**Study design:**

A cross-sectional study based on data from Electronic Medical Records (EMR).

**Methods:**

All children born in 2005 with permanent address in Greenland at the time of data extraction with a registered weight and height in EMR from January 1st 2011 to January 31st 2013 were included in the study. Information about height without shoes and weight in light indoor clothing was obtained. Body Mass Index (BMI) was calculated. Participants were categorized into age and gender-specific weight classes based on the International Obesity Task Force (IOTF) cut-offs for child overweight, obesity and thinness.

**Results:**

A total of 842 children born in 2005 were identified. Of those, 72% (N=607, 308 boys and 299 girls) had a recorded weight and height in the study period. In total, 74.6% (71.2–78.1) were categorized as of normal weight. The proportion of children with overweight was 15.8% (12.9–18.7) while 6.8% (4.8–8.8) were obese. In all, 2.9% were categorized as thin. The proportion of overweight among boys (12.7%) was lower (p=0.031) than among girls (19.1%), and boys in Nuuk had a lower median BMI compared to the rest of Greenland. No differences in distribution of age and gender-specific overweight and obesity were observed between the capital and the rest of Greenland.

**Conclusion:**

Nearly 1 quarter of Greenlandic children are overweight or obese at school entry. No differences were observed between Nuuk and the rest of Greenland. Information about weight and height is available in the EMR for the majority of all children at school entry in Greenland. Continuous monitoring of the proportion of overweight and obesity among children using data from the EMR in Greenland is recommended.

Childhood obesity is a serious public health problem globally and its prevalence has increased to an alarming rate in many countries (1, 2). Also, among circumpolar Inuits, overweight and obesity has been documented to be on the increase during the past decades both among adults and children (3–7).

Greenland is the world's largest island situated in the North Atlantic Ocean between Europe and Canada. It is populated only along coastal areas and mainly on the west coast. Transportation is by air or by sea. The total population is about 56,300. One quarter of the population of Greenland live in the capital, Nuuk, a city of about 16.5 thousand inhabitants. The socio-economic development in Nuuk is generally higher than in the rest of Greenland, as the administration and most large institutions are placed here. In other parts of Greenland, 15 smaller towns and about 60 settlements are scattered and only 4 other towns consist of populations above 3,000 inhabitants.

In Nuuk a more than 2-fold increase in overweight and obesity has been documented in the 30 years from 1972 to 2002 with an increase from 9.6% to 22.5% of all children at school entry (6). However, it is unknown if the prevalence of overweight and obesity among children in Nuuk has continued to rise in the past 10 years. It is also unknown if the prevalence of overweight and obesity in Nuuk reflects the prevalence in all of Greenland since Nuuk as a capital has a more well-educated, affluent population with a more westernized life style, and due to its larger size, the population has access to more health care services, more different foods and more recreational activities compared to the rest of Greenland. In addition, approximately 17% of the study population in the capital is born outside Greenland compared to around 4% in the rest of Greenland leaving a possibility of ethnical and cultural differences (8).

Obesity is associated with increased morbidity and mortality in children as well as in adults (1). Prevention is crucial. Once obesity is attained, it is difficult to treat with permanent results although lifestyle programmes can reduce the level of overweight in child and adolescent obesity, 6 and 12 months after the beginning of the programme (9). Still information on the long-term outcome of obesity treatment in children and adolescents is limited (9). Also, in Greenland, childhood obesity and overweight at school entry has been found as a strong predictor for adolescent overweight and obesity (6).

Implementation of on-going extraction of weight and height among children through the EMR in Greenland might make it possible to visualize trends and evolution of overweight and obesity in the country over a shorter or longer period of time in a feasible and easy way, although the method remains unexplored.

The objective of this study was to estimate the proportion of gender-specific thinness, overweight and obesity among children born in 2005 at school entry in Greenland using the EMR and compare these figures between the capital, Nuuk, and the rest of Greenland.

## Methods

A free and mandatory school system is in use throughout Greenland. Children start school in the year they turn 6. All children must according to regulations be offered a routine health examination within the first 2 years of school attendance. Since 2007, Electronic Medical Records (EMR) was implemented throughout Greenland. Data from school health examinations was included in the EMR in 2011. All children born in 2005 with a permanent address in Greenland and an EMR registered weight and height within the past 25 months from January 1st 2011 to January 31st 2013 were included in the study. Only 1 weight and 1 height measurement for each child was present in the EMR as a result of data not being implemented before 2011. Their permanent address was affiliated to one of the 16 districts.

Information about gender, age at examinations time, height without shoes and weight in light indoor clothing was obtained. The study was approved by the ethical commission for health research in Greenland (Reference number 2013–081849). Body Mass Index (BMI) was calculated as kg/m^2^. Participants were categorized into age and gender-specific weight classes based on the IOTF cut-offs for child overweight, obesity and thinness (10, 11), which permits comparison of results with earlier studies from Greenland (6, 12, 13), Canada (14, 15) and Denmark (16). Thinness grade III corresponds to BMI below 16 kg/m^2^, thinness grade II to BMI below 17 kg/m^2^ and thinness grade I to BMI below 18.5 kg/m^2^ at age 18 years while overweight BMI corresponds to BMI above 25 kg/m^2^ and obesity BMI corresponds to BMI above 30 kg/m^2^ at age 18 years. A sub-analysis including review of the medical records in children in Nuuk with no weight registered in the EMR was performed to find the reason for their non-participation.

Variables were described using medians and inter quartile range (IQR). Proportions were calculated with 95% confidence intervals (95% CI). Medians were analyzed using Mann–Whitney U-test while proportions were compared using Chi square test. P-value below 0.05 was used as significance level.

## Results

A total of 842 children born in 2005 with residence in Greenland were identified. Of those, 72% (N=607, 308 boys and 299 girls, range 5.1–8.0 years) had a recorded weight and height in the study period. The missing 28% (233 children) were either not examined or not registered correctly in the EMR. The proportion of investigated children varied among districts. In Nuuk, 221 children were registered, and data were found on 155 corresponding to 70%. In Aasiaat, 55 children were examined (90%), while the same figures were 65 (87%) for Ilulissat, 35 (81%) for Maniitsoq, 20 (83%) for Nanortalik, 17 (65%) for Narsaq, 3 (11%) for Paamiut, 10 (22%) for Qaqortoq, 5 (29%) for Qasigiannguit, 16 (70%) for Qeqertarsuaq, 81 (74%) for Sisimiut, 24 (77%) for Uummannaq, 5 (100%) for Illoqqortoormiut, 14 (93%) for Qaanaaq, 61 (88%) for Tasiilaq and 43 (84%) for Upernavik. The sub-analysis included the 66 of 221 children (30%) in Nuuk with no weight registered in the EMR showing that 38 children were new settlers or children that had moved from Nuuk temporarily, while 28 refrained from attending.

Age, height, weight and BMI for boys and girls in Nuuk and for the rest of Greenland are listed in Table I. Boys in Nuuk had a lower median BMI than boys in the rest of Greenland (p=0.013). No other differences between Nuuk and the rest of Greenland were observed.

**Table I:** Distribution age, height, weight and BMI among boys and girls in Nuuk and the rest of Greenland

	Male (N=308)			Female (N=299)			Total (N=607)		
			
	Nuuk (N=79)	Rest of Greenland (N=229)	p	Nuuk (N=76)	Rest of Greenland (N=223)	p	Nuuk (N=155)	Rest of Greenland (N=452)	p
	Median (IQR)	Median (IQR)		Median (IQR)	Median (IQR)		Median (IQR)	Median (IQR)	
Age (years)	6.5 (0.5)	6.6 (0.5)	0.070	6.6 (0.6)	6.5 (0.7)	0.441	6.5 (0.5)	6.6 (0.6)	0.179
Weight (kg)	23.5 (4.0)	24.1 (4.7)	0.174	24.7 (4.55)	23.6 (4.6)	0.78	24.0 (4.5)	24.0 (4.6)	0.751
Height (m)	1.23 (0.06)	1.22 (0.3)	0.639	1.21 (0.06)	1.20 (0.08)	0.219	1.22 (0.06)	1.21 (0.08)	0.493
BMI (kg/m^2^)	16.0 (1.9)	16.6 (1.8)	**0.013**	16.7 (2.9)	16.3 (2.1)	0.267	16.2 (2.3)	16.5 (2.0)	0.218

Medians analyzed using Mann–Whitney U-test. Proportions were compared using Chi square test. P-value below 0.05 was used as significance level. Significant values are presented in bold. IQR: Inter quartile range; BMI: body mass index.

Girls had a lower median height (1.20 m) at examination than boys (1.22 m) (p=0.016). No differences in age, weight or BMI were observed between genders (data not shown).

Distribution of weight classes for boys and girls in all of Greenland are shown in Table II. In total, 74.6% were categorized as normal weight. In all, 22.6% were above normal weight while 2.8% were categorized as thin. The proportion of boys with overweight was 12.7%, which was significantly (p=0.031) lower than the proportion of girls with overweight 19.1%. No differences in the distribution of weight classes were observed between Nuuk and the rest of Greenland neither for boys or girls (see Table III).

**Table II:** Distribution of weight classes among Greenlandic boys and girls at school entry

	Male (N=308)	Female (N=299)	p	Total (N=607)
Weight class	% (CI 95)	% (CI 95)		% (CI 95)
Thinness grade 1–3	2.3 (0.6–3.9)	3.3 (1.3–5.4)	0.424	2.8 (1.5–4.1)
Normal weight	79.2 (74.7–83.8)	69.9 (64.7–75.1)	**0.008**	74.6 (71.2–78.1)
Overweight	12.7 (8.9–16.4)	19.1 (14.6–23.5)	**0.031**	15.8 (12.9–18.7)
Obesity	5.8 (3.2–8.5)	7.7 (4.7–10.7)	0.36	6.8 (4.8–8.8)

Proportions compared using Chi square test. P-value below 0.05 was used as significance level. Significant values are presented in bold. Thinness grade III corresponds to BMI below 16 kg/m^2^, thinness grade II to BMI below 17 kg/m^2^ and thinness grade I to BMI below 18.5 kg/m^2^ at age 18 years while overweight BMI corresponds to BMI above 25 kg/m^2^ and obesity BMI to BMI above 30 kg/m^2^ at age 18 years. CI 95: 95% confidence intervals.

**Table III:** Distribution of weight classes in Nuuk and in the rest of Greenland

	Male (N=308)			Female (N=299)			Total (N=607)		
			
Weight class	Nuuk (N=79)	Rest of Greenland (N=229)	p	Nuuk (N=76)	Rest of Greenland (N=223)	p	Nuuk (N=155)	Rest of Greenland (N=452)	p
	% (CI 95)	% (CI 95)		% (CI 95)	% (CI 95)		% (CI 95)	% (CI 95)	
Thinness grade 1–3	3.8 (−0.4–8.0)	1.7 (0.0–3.4)	NA	1.3 (−0.2–2.9)	3.1 (0.9–5.4)	0.29	3.9 (0.8–6.9)	2.4 (0.1–3.9)	0.35
Normal weight	84.8 (76.9–92.7)	77.3 (71.9–82.7)	0.16	67.1 (56.5–77.7)	70.9 (64.9–76.8)	0.54	76.1 (69.4–82.8)	74.1 (70.1–78.2)	0.62
Overweight	8.9 (2.6–15.1)	14.0 (9.5–18.5)	0.24	17.1 (8.6–25.6)	19.7 (14.5–25.0)	0.62	12.9 (7.6–18.2)	16.8 (13.4–20.3)	0.25
Obese	2.5 (−0.9–6.0)	7.0 (3.7–10.3)	0.15	11.8 (4.6–19.1)	6.3 (3.1–9.5)	0.12	7.1 (3.1–11.1)	6.6 (4.3–8.9)	0.84

Proportions were compared using Chi square test. P-value below 0.05 was used as significance level. Thinness grade III corresponds to BMI below 16 kg/m^2^, thinness grade II to BMI below 17 kg/m^2^ and thinness grade I to BMI below 18.5 kg/m^2^ at age 18 years while overweight BMI corresponds to BMI above 25 kg/m^2^ and obesity BMI corresponds to BMI above 30 kg/m^2^ at age 18 years. CI 95: 95% confidence intervals; NA: Data not applicable.

## Discussion

This study showed that the majority (74.6%) of children in Greenland born in 2005 had a normal weight at school entry. The proportion of children with overweight was 15.8% (12.9–18.7) while 6.8% (4.8–8.8) were obese. Only 2.8% (1.5–4.1) were categorized as thin. More girls than boys were overweight. No differences between Nuuk and the rest of Greenland were observed.

According to Schnohr, Niclasen et al. (6, 12, 13), overweight and obesity at school entry in Nuuk has been on the increase since the 1970s with around 9% overweight and 0.8% obese up until the 1990s with 17% overweight and 5% obesity (1992–1996). Hereafter, the proportion of overweight was steady around 17% and obesity around 5–6% in the 1990s. Based on the same BMI cut-offs (10), our data showed a prevalence in Nuuk of overweight of 12.9% and for obesity 7.1%. This indicates that the total amount of children above normal weight is stable around 20 to 25% or might even be decreasing. The proportion of obesity may be increasing. The proportion of overweight is comparable to the Danish ones. Thus, a Danish study reported 16.6% overweight and 4.2% to be obese among Danish children at school entry (17).

In our study, significantly more girls were overweight (19.1%) than boys (12.7%) (p=0.031). The same trend has been reported in a Danish cohort study among children in Copenhagen. They reported 21% overweight and 4% obesity among girls. Among boys 15% were overweight and 5% obese (16).

This pattern has also been reported among adult Greenlanders (4, 18, 19). Earlier studies among adult Canadians and Alaskan Inuits have shown that women have a higher prevalence of obesity, but lower prevalence of overweight than men (20, 21).

Among adult Danes, overweight and obesity is more pronounced among men (22).

Even if about 1 quarter of Greenlandic children is overweight or obese, the figures are not as alarming as in other Inuit societies. Egeland, Galloway and Johnson-Down et al. (14, 15, 23) used data derived from the Nunavut Inuit Child Health Survey 2007–2008, including 388 or 26% of the 3- to 5-year-old Inuit population. Based on both the CDC (24) and the IOTF/Cole 2000 standard (10) on BMI in children, 67% of the 5-year-olds were found overweight (39%) or obese (28%). The authors related overweight and obesity to the very high frequency of food insecurity found (14). The relationship between food insecurity and obesity is still debated (25). Still, it might be at least 1 explanatory factor in young Inuits as studies have revealed a much lower frequency of food insecurity in Greenland (26). Both the closer resemblance between BMI at school entry between Danish and Greenlandic children than between Greenlandic children and other Inuits, and the lower proportion of food insecurity found in Greenland indicate environmental and societal organization as important factors for the development of obesity. In Greenland, the provision of basic healthy food is considered to be a governmental responsibility. Healthy foods are subsidized and made available to the population in remote areas through publicly owned stores. It secures basic foods to prices resembling town level, even if the variety of basic foods provided depends on the actual population size. In Canada and the United States, a more complex pattern of subsidized market mechanisms and charity exists (27–29). In contrast, studies among adult Inuits in Greenland and Canada show that affluence has been associated with obesity for both men and women (4, 20).

While overweight and obesity is found related to low income and low education in high-income western countries (2), it has been found related to high affluence in many developing countries (2, 30). In adults, obesity still has been found positively related to affluence among Greenlanders (4, 31) while in this study boys from other parts of Greenland had a higher median BMI than boys in Nuuk (p=0.013) (Table I).

Earlier, overweight may have been considered as positive by Greenlanders, indicating culturally that the family was well supplied. If this point of view exists among groups of Greenlanders today it has not yet been proven through research, yet overweight is not always considered undesirable (32) and people's perceptions of body image and BMI among children could be different between Nuuk and other parts of Greenland, affected by traditional Greenlandic culture, interference of other ethnic cultures and difference in socio-economic structure.

### Strength and weaknesses

The major strength of the study is that data were collected nationally and could be identified electronically. The main limitation is that only 1 birth year is represented limiting the statistical power of the study. However, we could not include earlier data since the electronic registration of weight and height was not implemented nationally before 2011. Even in this study, only 72% of all children were represented. While the drop-out in Nuuk was caused by migration and by non-attendance, discrepancy in the registration practice seems to exist in the rest of Greenland.

Measurements of weight and height may slightly differ between observers, although systematic bias is unlikely. Due to only having a single measure of weight and height for each child, inter- and intra-observer error could not be calculated.

No socio-economic or lifestyle variables were used nor ethnic origin.

BMI is an index of weight to height (kg/m^2^). While it is not a direct measure of body fat or lean tissue, it is the most commonly used indicator of health risks associated with overweight such as type 2 diabetes mellitus, insulin resistance and cardiovascular disease and underweight such as osteoporosis and infertility in adults (33). For children BMI varies strongly by gender and age.

An overestimation of BMI for Inuits due to shorter extremities compared to other races has also been suggested (33, 34). A 10% overestimation of BMI in adult Inuits compared to Caucasians has been proposed (34). Still, BMI is useful for predicting overweight at the population level, and BMI is a widely used as an indirect measurement of overweight and obesity in childhood due to its feasibility, practicality and non-invasiveness (17, 35). Despite its limitations and for reason of direct comparison with studies among Greenlandics, other Inuits and Danish children, we used BMI and the Cole 2000 Standard (10) for overweight and obesity BMI cut-offs for children.

Although age-specific BMI cut-offs were used, the few 5-, 7- and 8-year-old children examined may reduce the comparability of the results and may introduce potential bias as outliers.

### Conclusion and perspective

Potential interventions of obesity in youth span a continuum from preventing the development of obesity to treating established obesity and its complications, but tailoring preventive programmes requires knowledge on the distribution and tracking of weight in children. This study demonstrates that extraction of data from the EMR could be a feasible way to monitor childhood BMI and BMI subgroups at school entry in Greenland prospectively. Correct registry of height and weight in the EMR is recommended for easy and universal data extraction. We recommend the BMI at school entry as an indicator of early childhood thinness, overweight and obesity in Greenland. Further research on childhood overweight and obesity in Greenland is needed to enlighten the found gender difference, health aspects and toassess future differences in the development across the country.
